# Use of phage display biopanning as a tool to design CAR-T cells against glioma stem cells

**DOI:** 10.3389/fonc.2023.1124272

**Published:** 2023-03-24

**Authors:** Marine Potez, Sebastian Snedal, Chunhua She, Jongmyung Kim, Konrad Thorner, Timothy H. Tran, Maria Cecilia Ramello, Daniel Abate-Daga, James K. C. Liu

**Affiliations:** ^1^ Neurosurgical Oncology, Department of Neuro-Oncology, H. Lee Moffitt Cancer Center and Research Institute, Tampa, FL, United States; ^2^ Department of Immunology, H. Lee Moffitt Cancer Center and Research Institute, Tampa, FL, United States; ^3^ Chemical Biology Core, H. Lee Moffitt Cancer Center and Research Institute, Tampa, FL, United States; ^4^ Department of Oncologic Sciences, Morsani College of Medicine, University of South Florida, Tampa, FL, United States

**Keywords:** glioblastoma, glioma stem cells, novel binding domains, phage display biopanning, CAR-T, peptides, N-cadherin

## Abstract

**Background:**

Glioblastoma (GBM) is both the most common and aggressive type of primary brain tumor, associated with high mortality rates and resistance to conventional therapy. Despite recent advancements in knowledge and molecular profiling, recurrence of GBM is nearly inevitable. This recurrence has been attributed to the presence of glioma stem cells (GSCs), a small fraction of cells resistant to standard-of-care treatments and capable of self-renewal and tumor initiation. Therefore, targeting these cancer stem cells will allow for the development of more effective therapeutic strategies against GBM. We have previously identified several 7-amino acid length peptides which specifically target GSCs through *in vitro* and *in vivo* phage display biopanning.

**Methods and results:**

We have combined two of these peptides to create a dual peptide construct (EV), and demonstrated its ability to bind GSCs *in vitro* and target intracranial GBM in mouse models. A peptide pull-down performed with peptide EV followed by mass spectrometry determined N-cadherin as the binding partner of the peptide, which was validated by enzyme-linked immunosorbent assay and surface plasmon resonance. To develop cytotoxic cellular products aimed at specifically targeting GSCs, chimeric antigen receptors (CARs) were engineered containing the peptide EV in place of the single-chain variable fragment (scFv) as the antigen-binding domain. EV CAR-transduced T cells demonstrated specific reactivity towards GSCs by production of interferon-gamma when exposed to GSCs, in addition to the induction of GSC-specific apoptosis as illustrated by Annexin-V staining.

**Conclusion:**

These results exemplify the use of phage display biopanning for the isolation of GSC-targeting peptides, and their potential application in the development of novel cytotoxic therapies for GBM.

## Introduction

Glioblastoma (GBM) is the most common and malignant form of primary brain tumor and is associated with a poor prognosis with a median survival time of 16 months (1). The current standard-of-care treatment consists of maximal-safe surgical resection, followed by concurrent chemotherapy and radiotherapy, and maintenance chemotherapy (2). Recurrence is nearly inevitable, and less than 5% of patients survive 5 years after diagnosis (3). The resistance of GBM to conventional treatments remains an elusive challenge of this cancer, highlighting the need for novel therapeutic approaches.

*Ex-vivo* expansion of T cells followed by genetic modification with a chimeric antigen receptor (CAR) is used to redirect the immune cells to target various types of tumor cells. A CAR is a synthetic protein that typically consists of an extracellular antigen-recognition domain that contains the heavy and light chain variable fragments of a monoclonal antibody or B cell receptor, joined to a hinge and transmembrane domain, and an intracellular CD3ζ signaling domain from the T cell receptor, often in addition to a costimulatory domain such as CD28 (4). CAR-T cell activation and proliferation occur upon binding to their respective surface-exposed tumor antigen, thus leading to a cytotoxic attack on the antigen-bearing tumor cell (5). The clinical feasibility and safety of CAR-T cell therapy for patients with GBM has been demonstrated on three targets, interleukin-13 receptor alpha 2 (IL13-Rα2), human epidermal growth factor receptor 2 (HER2), and epidermal growth factor receptor variant III (EGFRvIII) (6–8). However, the clinical outcome remains unsatisfactory, with only partial responses being reported and a median survival time ranging between 8 and 11 months (9).

Tumor heterogeneity presently remains one of the main reasons for GBM resistance to treatment, largely due to the presence of cancer stem cells. Glioblastoma stem cells (GSCs) comprise a subpopulation of tumor cells that possess the unique abilities of self-renewal and tumor recapitulation, in addition to resistance to conventional radiotherapy and chemotherapy (10–12). Additional characteristics of these cells are the generation of differentiated progeny, invasive potential, and secretion of angiogenic factors (13–15). Several studies have identified and explored a variety of different markers of GSCs, such as CD133, CD15/SSEA, CD44, A2B5, SOX2, nestin, and OLIG2, however, none are able to universally define a GSC population (14, 16–20). Identification of a more ubiquitous GSC marker may allow for improved strategies to target and eliminate highly resistant tumors.

Phage display biopanning is a discovery tool that employs bacteriophages in which genes are inserted, and then displayed on their surface. Phage display allows for the isolation of peptides that can bind a specific cell type or protein through a series of positive and negative selection steps screening a library of random peptide sequences (21). The use of phage display biopanning to isolate peptides with specificity for GSCs has previously been employed (22, 23). Using a combination of 2 peptides previously isolated from phage display biopanning, we placed the dual peptide construct in the place of the scFv antigen-binding domain of a CAR, with the goal of designing a GSC-targeting cellular product. The peptide-based CAR-T cells were then evaluated for their reactivity and cytotoxicity.

## Materials and methods

### Approval and ethics statement

Experiments performed were approved by the Institutional Reviewed Board at the H. Lee Moffitt Cancer Center and Research Institute in accordance with the ethical guidelines set forth at each institution. GSCs, tumor tissue, and non-malignant normal brain were harvested from patient tumor samples or epileptic brain at Duke University Medical Center, Cleveland Clinic, and University Hospital Cleveland Medical Center in accordance with an approved protocol by the respective Institutional Review Boards. All animal studies were performed according to guidelines under Institutional Animal Care and Use Committee protocol approved at H. Lee Moffitt Cancer Center and Research Institute (IACUC# RIS00010727). Cg-Prkdcscid Il2rgtm1Wjl/SzJ (NSG) mice (male, 8 weeks old) were acquired from Jackson Laboratories (ME, USA), housed with ad libitum access to standard laboratory chow and water and with a 12-hour light/dark cycle.

### Patient specimens and cell culture

T387 and T4121 glioblastoma stem cells were obtained from tissue dissociation of patient derived glioblastoma samples and maintained as previously described (13, 23). Both cell lines were pathologically described as glioblastoma at the time of resection from the patient. T4121 was a recurrent GBM. No additional molecular identifiers were available. To obtain differentiated glioma cells (DGCs), neurospheres were grown in DMEM containing 10% FBS and 1% Penicillin-Streptomycin. A172 glioblastoma cells (ATCC^®^ CRL-1620™, Manassas, VA) and non-malignant brain cells (NM263) obtained from epileptic brain tissue were also maintained in DMEM supplemented by 10% FBS and 1% PS. Neural stem cells (NSCs) were obtained from EMD (Millipore, Temecula, CA) and cultured as described by the manufacturer. Human primary peripheral blood mononuclear cells (PBMCs) were obtained from de-identified buffy coats (OneBlood, Florida Blood Services, FL), cultured in X-VIVO medium (Lonza, Walkersville, MD) supplemented with 5% human serum (Access Biologicals, Vista, CA), 1% PS, 1% 200 mM L-glutamine, and 300 IU/mL of IL-2 (Clinigen, Yardley, PA), and activated with the anti-CD3ϵ monoclonal antibody OKT3 (Biolegend, San Diego, CA) for two days prior to transduction.

### *In vitro* and *in vivo* phage display biopanning

*In vitro* and *in vivo* phage display biopanning was performed using a Ph.D.-7 Phage Display Peptide Library Kit (New England BioLabs, Ipswich, MA). The first step of the *in vitro* biopanning was a negative selection of the library against extracellular matrix and DGCs to remove non-GSC binding peptides. The peptide library was then screened against GSCs for four rounds of biopanning. Phage clones were then isolated through bacterial infection and peptide sequences were isolated by sequencing. *In vivo* biopanning was performed by intravenously injecting the phage library in NSG mice bearing intracranial GBM. Following circulation of the peptide library for 24 hours, tumors were harvested, tumor cells were lysed, then bound phage peptides were isolated, purified, and transduced into E. coli for phage clone isolation and sequenced. The *in vivo* phage display performed against subcutaneous GBM was performed as previously described (22).

### Peptides

The peptide AWEFYFPGGGGSGGGGSGGGGSSSQPFWS was conjugated with cyanine 3 (EV-Cy3) or with biotin (EV-b), and the scrambled non-targeting peptides FAYPEWFGGGGSGGGGSGGGGSPSWSFSQ (NT-Cy3 and NT-b) were synthesized by LifeTein, LCC (Hillsborough, NJ, USA). They were reconstituted in DMSO at a concentration of 10 mM and stored at -80°C.

### Immunocytochemistry

After coating glass coverslips with Geltrex (1/100, Gibco, Waltham, MA) for 24 hours, cells were seeded and incubated overnight on the coated coverslips. The peptide cell staining was performed by removing the media and incubating the cells with PBS-1%-BSA for 15 minutes at 4°C. Biotinylated peptides (10 μM, EV-b or NT-b) were incubated for 20 minutes at 4°C, washed with PBS-1%-BSA for 5 minutes, and fluorescent-labeled streptavidin (1/100, AlexaFluor 647, Invitrogen, Waltham, MA) was added and incubated for 20 minutes at 4°C. The cells were then washed (PBS-1%-BSA, 5 minutes), fixed with 4% paraformaldehyde (20 minutes, RT), incubated with a 4’,6-diamidino-2-phenylindole (DAPI) solution (PBS-1%-BSA + 100 ng/mL DAPI, 20 minutes, RT) and mounted with Anti-Fade Fluorescence Mounting Medium (Abcam, Cambridge, UK). Z-stack images were acquired with a Leica SP8 confocal microscope at 63 magnification 1X and 3X. All images were post-processed identically with Image J software (Bethesda, MD).

### Flow cytometry

For the peptide-binding quantification and binding affinity assays, single cell suspensions of GSCs or DGCs (1×10^6^ cells/mL) were saturated with PBS-1%-BSA for 15 minutes on ice, centrifuged at 150 x g for 3 minutes, then incubated at 4°C for 30 minutes with 10 μM or increased concentration of biotinylated peptide (EV-b or NT-b). Cells were washed two times and incubated with an AlexaFluor 647-streptavidin (1/100, Invitrogen, Waltham, MA) at 4°C for 30 minutes in the dark. Cells were washed two times with PBS-1%-BSA and resuspended in 50 μL of PBS-1%-BSA with DAPI (100 ng/mL, Invitrogen, Waltham, MA). An Amnis ImageStream flow cytometer (Luminex, Austin, TX) was used for recording and data was analyzed with Image Data Exploration and Analysis Software (IDEAS, EMD Millipore, Burlington, MA). Gating strategy shown in **Supplemental Figure 1**.

For the quantification of CAR transduction efficiency, T cells co-transduced with the EV CAR and truncated CD34, and untransduced (UT) T cells were washed, stained with CD34-PE (Invitrogen, MA5-16927), then incubated for 15 minutes at 4°C in the dark. Cells were then washed and resuspended in FACS buffer with DAPI and incubated for 15 minutes at 4°C in the dark. Data acquisition was performed with a BD FACSCanto II flow cytometer (Becton, Dickinson & Company, Franklin Lakes, NJ).

For apoptosis assay experiments, single cell suspensions of effector and target cells (4×10^5^ and 8×10^5^ cells, respectively) were surface-stained with CSPG4-APC (R&D Systems, FAB2585A), and CD3-BV711 (Biolegend, 317328), following a 4-6 hour co-culture, then incubated for 20 minutes at 4°C in the dark. Cells were then washed, secondarily stained with Annexin-V-FITC (Biolegend, 640906) and DAPI, incubated for 15 minutes at room temperature in the dark, and then analyzed. Data acquisition was performed with BD FACSCanto II or LSR II cytometers (Becton, Dickinson & Company, Franklin Lakes, NJ) and analyzed with FlowJo software (Becton, Dickinson & Company, Ashland, OR). Gating strategy shown in **Supplemental Figure 6**.

### *In vivo* peptide injection

For *in vivo* imaging, a stereotaxic implantation was performed to inject 5 μL with 5×10^5^ glioma stem cells transduced with luciferase, or 5 μL of PBS for the sham mice, in the right caudate nucleus with a Hamilton syringe. Two weeks after tumor implantation, 20 μM of EV-b stained by streptavidin-Alexa-Fluor 647 (1/100) was injected intravenously in the tail vein of the mice and intraperitoneal injection of 100 μL Luciferin (15 mg/mL, GoldBio, St Louis, MO) was performed to confirm the presence of GBM. Images were acquired on an IVIS Lumina (PerkinElmer, Waltham, MA) with excitation and emission filters of 660 and 710 nm, respectively, and a luminescence filter for luciferase cells. Measurements were performed by subtracting the fluorescence before injection from the fluorescence after injection on the lower back, and on the head.

### Peptide pull-down assay

Cells were lysed using the IP Lysis Buffer Protocol (Pierce, Waltham, MA) as described by the manufacturer. Biotinylated peptides (EV-b and NT-b, 40 μg) were incubated with 100 μL of streptavidin agarose resin at 4°C for 1 hour on a rotator to generate the peptide-coupled streptavidin agarose resin. The protein samples were pre-cleared by incubation of 50 μL of NT-streptavidin agarose resins (4°C on rotator for 1 hour). After centrifugation, the pre-cleared samples were incubated with 150 μL of EV-coupled streptavidin agarose resin at 4°C overnight on rotator. After PBS wash, the resin was pelleted and sent for mass spectrometry analysis (see details in **Supplementary Methods**) or eluted with 4x Laemmli Sample Buffer (Bio-Rad, Hercules, CA) containing 1X DTT reducing agent (dithiothreitol, Cell Signaling Technology, Danvers, MA) and boiled for SDS-PAGE. Pulled-down proteins were then separated with a Novex™ WedgeWell™ (ThermoFisher Scientific, Waltham, MA) and transferred to polyvinylidene fluoride (PVDF) membranes (Millipore, Burlington, MA). Membrane-transferred proteins were immunoblotted with an antibody recognizing N-cadherin (1/10000, NBP2-01498, Novus Biologicals, Littleton, CO) (**Supplemental Figure 2**).

### Peptide-protein interaction profiling on HuProt™ arrays

The binding profile of EV-Cy3 peptide was evaluated on HuProt™ human proteome arrays, a human protein collection on a single array. The samples were sent to CDI Laboratories (Baltimore, MD) for the profiling assay of the samples. Candidates were identified using the following criteria: (1) The mean signal intensity of the sample group (EV-Cy3) is greater than 1.25-fold of the NT-Cy3 group; (2) p value < 0.05 (t-test); (3) the signal intensity of the candidates is at least three standard deviations above the mean signal intensity of the sample group (EV-Cy3).

### Surface plasmon resonance

The direct binding measurements between N-cadherin and EV peptide by surface plasmon resonance was performed by immobilizing N-cadherin protein (Recombinant Human N-Cadherin extracellular domain, amino acids 160-724, 1388NC050, R&D Systems, Minneapolis, MN) to a CM5 sensor chip surface docked in Biacore T200 (Cytiva, Marlborough, MA) at 25°C using an amine-coupling method. Prior to immobilization, the chip was primed and equilibrated with a running buffer containing 20 mM HEPES, pH 7.4, 1 mM TCEP, 2 mM CaCl_2_ and 150 mM NaCl at a flow rate of 10 µL/min. The carboxymethyl surface of the CM5 chip was manually activated for 7 minutes using a 1:1 ratio of 0.4 M 1-ethyl-3-(3-diaminopropyl) carbodiimide hydrochloride (EDC) and 0.1 M N-hydroxysuccinimide (NHS) to achieve a density of about 10,000 RU per flow cell. N-cadherin protein stock (1.12 µM) was diluted to 100 nM in 10 mM sodium acetate, pH 4.5, and manually injected over the surface at the same flow rate. Excess activated functional groups on the surface were blocked using a 7-minute injection of 1M ethanolamine, pH 8.5, at a flow rate of 10 µL/min. Using this manual injection protocol, approximately 3500 RU of N-cadherin was immobilized on the surface of the CM5 chip.

For kinetic titration experiments, the same running buffer was used except with the inclusion of 5% DMSO. SPR single cycle kinetic experiments with EV peptide were carried out in duplicate on two different sensor chips at 25°C. A threefold concentration series of EV peptide serially diluted in the running buffer ranging from 2 to 167 µM was injected over the sensor surface at 30 µL/min flow rate with 80-second contact time and 120-second dissociating time. For control experiments, a non-targeting (NT) peptide (GGGSGGG) was applied in place of EV peptide under identical condition. Sensorgrams were solvent corrected, buffer referenced, and the on-rate, off-rate, and equilibrium binding constants were calculated from the sensorgrams by global fitting of a 1:1 binding model, using analysis software (version 3.0) provided with Biacore T200 instrument (Cytiva).

### Western blotting

T cells (5×10^6^ per effector condition) were lysed with Laemmli buffer, and protein concentrations were quantified utilizing the Pierce BCA Protein Assay Kit (ThermoScientific, Waltham, MA). Membranes were incubated with 0.2 μg/mL mouse anti-human CD3ζ (Santa Cruz Biotechnology, SC-166275, Dallas, TX) and 0.04 μg/mL rabbit anti-human glyceraldehyde-3-phosphate dehydrogenase (Santa Cruz Biotechnology, SC-25778, Dallas, TX). Membranes were then washed and subsequently incubated with 0.05 μg/mL of corresponding secondary antibodies, goat anti-rabbit 680LT (LI-COR, 926-68021, Lincoln, NE), and goat anti-mouse 800CW (LI-COR, 32210, Lincoln, NE) in 5% blocking solution for 1 hour. After washing and drying, the membrane blots were developed utilizing the LI-COR Odyssey imaging system with both the 700 and 800 channels (**Supplemental Figure 3**).

### Enzyme-linked immunosorbent assay

For evaluation of the peptide target, Nunc MaxiSorp, 96-well plates (ThermoFisher Scientific, Waltham, MA) were coated with 2.5 μg/mL of recombinant N-cadherin (Recombinant Human N-Cadherin extracellular domain, amino acids 160-724, 1388NC050, R&D Systems, Minneapolis, MN) diluted in 0.1 M NaHCO_3_ overnight at 4°C. The wells were washed with PBS-0.025%-Tween 20 and blocked with PBS/BSA-3%-Milk at RT for 2 hours. Biotinylated peptides (EV-b or NT-b), diluted in PBS-2%-BSA at different concentrations, were incubated for 1 hour at RT and washed five times. The streptavidin-HRP (0.1 μg/mL) was incubated for 1 hour at RT followed by five washes and incubation with 3,3′,5,5′-tetramethylbenzidine substrate (TMB, 50 μL/well, ThermoFisher Scientific, Waltham, MA) for 10 minutes at RT. 50 μL of 2 M sulfuric acid (H_2_SO_4_) were added to each well to stop the coloration and the absorbance was immediately read at 450 nm. Five replicates were used for each concentration, and wells without N-cadherin coating served as control.

For evaluation of IFN-gamma secretion in co-culture experiments, Nunc MaxiSorp 96-well plates were coated at RT overnight with 0.63 μg/mL of recombinant human IFN-gamma M700A monoclonal antibody (ThermoFisher, Waltham, MA) diluted with 1X PBS. Wells were washed with PBS-0.2%-Tween 20 and blocked with PBS-4%-BSA at RT for 1 hour. IFN-gamma recombinant protein was prepared in a 1:1000 ratio with PBS-4%-BSA for use as a serially diluted standard for later interpolating concentrations of IFN-gamma in pg/mL. Co-culture supernatants were prepared with PBS-4%-BSA in a 1:10 ratio, then added to the ELISA plates and incubated at RT for 1 hour. 0.16 μg/mL biotinylated recombinant IFN-gamma M701B monoclonal antibody (ThermoFisher, Waltham, MA) was prepared with PBS-4%-BSA, then added to the ELISA plates and incubated at RT for 1 hour. Precision Protein StrepTactin-HRP Conjugate (Bio-Rad, Hercules, CA) was prepared with PBS in a 1:5500 ratio, then added to the ELISA plates and incubated at RT for 30 minutes. 1-Step Ultra TMB Substrate (ThermoFisher, Waltham, MA) was added to the ELISA plates, then incubated for 10-15 minutes in the dark. Reactions were stopped by the addition of H_2_SO_4_, and the absorbance was immediately read at 450 nm and 550 nm. Experimental conditions were run in duplicate.

### Retroviral vectors and cell transduction

For CAR cloning, gBlock gene fragments were designed to contain the codon-optimized coding sequence for the indicated target-binding domains, and synthesized by Integrated DNA Technologies. These were flanked by NcoI/NotI restriction sites, which were used for cloning by restriction enzyme digestion followed by ligation. Using this approach, we replaced the scFv-encoding sequence of PSCA-28t28z and PSCA8t28z plasmids, described previously (24). Chemically competent *E. coli* (Invitrogen, Waltham, MA) were transformed for amplification of the CAR plasmids. Retroviruses encoding for our CAR constructs were generated utilizing 293GP cells. These packaging cells were co-transfected for 7 hours on poly-D-lysine-coated plates with RD-114 envelope protein in addition to the CAR plasmid and lipofectamine 2000 (Invitrogen, Waltham, MA). For the T cell transductions, RetroNectin-coated plates (Takara, Kusatsu, Shiga, Japan) were coated with retrovirus and centrifuged for 2 hours at 4000 x g at 32°C, after which the virus was aspirated and 2×10^6^ activated αβ T cells were seeded per well. The cells were centrifuged for 10 minutes at 1000 x g at 32°C, and then incubated for 24 hours before undergoing a second transduction.

### Statistics

All statistics were performed with GraphPad Prism 8.2 (San Diego, CA). ImageStream flow cytometry data was analyzed with a one-way ANOVA and a Dunnett’s multiple comparisons test. The binding affinities (Kd) were calculated with the One site-specific binding equation. A Kruskal-Wallis uncorrected test was used to compare the binding of EV and N-cadherin by ELISA. The difference between the *in vivo* fluorescence of the body and the head of the mice after EV injection was evaluated with a Wilcoxon paired test. IFN-gamma ELISA data was analyzed by two-way ANOVA and Tukey’s multiple comparisons test to determine significance between CAR and GFP conditions within the same target cell group, and to compare CAR conditions across the different targets. The Annexin-V apoptosis data was analyzed by two-way ANOVA and Sidak’s multiple comparisons test to determine significance between CAR and UT conditions against the target cell conditions. Proteome and mass spectrometry data was analyzed by T tests at Moffitt’s core facilities.

## Results

### Tandem sequences combining two GSC-specific peptides target GSCs *in vitro* and *in vivo*

**Figure 1:**
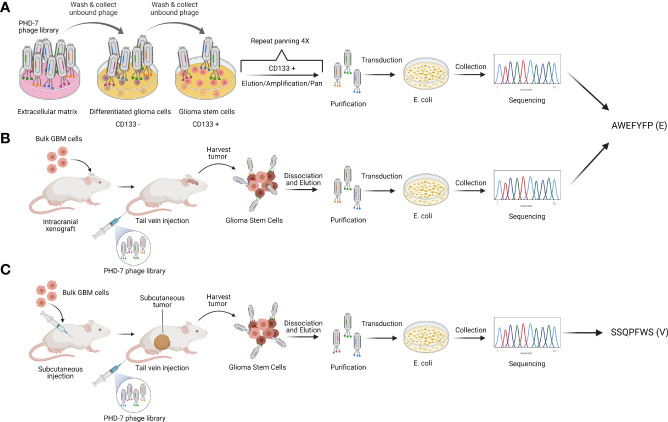
Peptide selection. Schematic representation of phage display strategies used to isolate the GSC-targeting peptides E (AWEFYFP) and V (SSQPFWS). **(A)** In vitro and **(B)** *in vivo* biopanning resulted in the identification of peptide E as one of the most enriched. **(C)** *In vivo* biopanning on subcutaneous tumors identified peptide V as one of the most enriched. Authors’ elaboration based on prior data from (22, 23).

We have previously isolated glioblastoma stem cell targeting peptides through a combination of *in vitro* and *in vivo* biopanning strategies (22, 23). *In vitro* biopanning was performed by screening a random 7 amino acid phage peptide library against differentiated stem cells and glioblastoma stem cells grown in culture to isolate GSC-specific targeting peptides (**Figure 1A**). Two types of *in vivo* biopanning were performed, one against mice with intracranial GBM xenografts (**Figure 1B**) and one with subcutaneous flank xenografts (**Figure 1C**). Two peptides were selected for use in subsequent experiments. A single peptide sequence AWEFYFP (E) was isolated from both the *in vitro* and intracranial *in vivo* biopanning. Another peptide, SSQPFWS (V), was isolated from the subcutaneous flank biopanning. These two peptides were previously demonstrated to have specificity for targeting GSCs *in vitro* and therefore used to design new CAR constructs.

**Figure 2:**
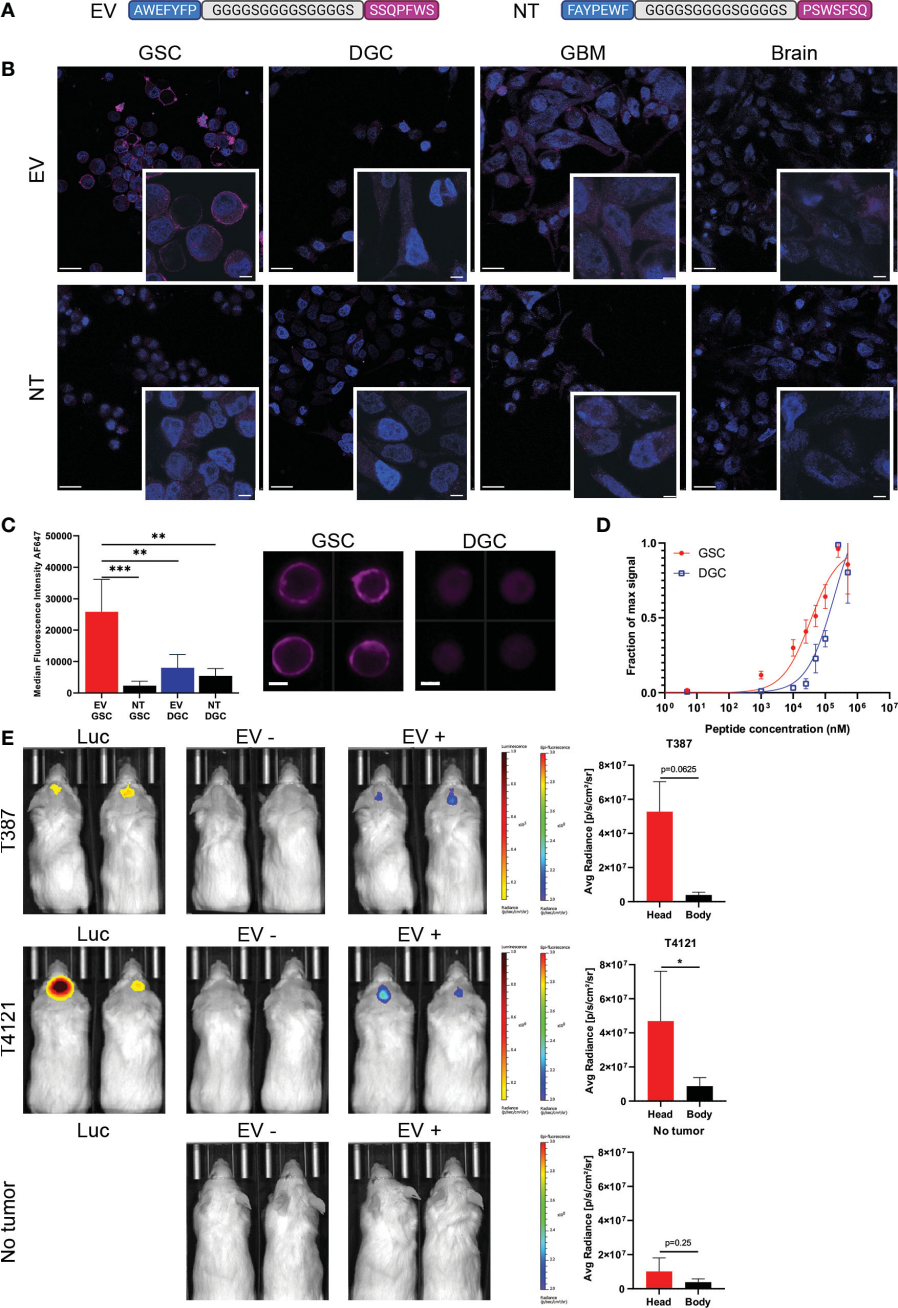
Specificity of the peptide for GSCs. **(A)** The two peptides recovered with phage display biopanning have been combined as a dual peptide (EV), linked by a sequence of glycine and serine. A non-targeting peptide (NT) employed the same amino acids in a scrambled sequence. **(B)** Immunocyto-fluorescent staining of different cells with EV or NT peptides (purple staining). Nuclei are stained with DAPI (blue staining). Scale bars: 20 μm, in zoomed images: 5 μm. **(C)** ImageStream quantification and representative images of EV staining on GSCs (red) and DGCs (blue), compared to NT (black bars), ** = p < 0.01, *** = p < 0.001. Signal intensity represented is actual measured median fluorescence intensity. Data in quadruplicate. Scale bars: 10μm. **(D)** Measure of the binding affinity of GSCs (red) or DGCs (blue) with increasing concentration of EV peptide by flow cytometry. Data in quadruplicate. **(E)** Representative images of mice and fluorescence measurements acquired with the IVIS Lumina. The presence of tumor was confirmed by luminescence after Luciferin injection. Images were acquired before (EV-) and after (EV+) EV injection for the T387 cells (top row), T4121 cells (middle row), and sham implanted (bottom row). The body measurements were taken at the lower back of the mice. The fluorescence scale remained the same for all the mice, and the luminescence scale was adapted between T387 and T4121 tumors, * = p < 0.05.

To combine the binding affinities, peptides AWEFYFP (E) and SSQPFWS (V) were joined by a flexible linker (GGGGSGGGGSGGGGS) resulting in a final length of 29 amino acids (EV). A scrambled sequence of both peptides joined by the same linker was used as a control peptide (NT) (**Figure 2A**). The EV peptide demonstrated specificity for binding GSCs compared to the NT peptide, and specificity over DGCs, GBM cell line, non-neoplastic brain cells (**Figure 2B**). Flow cytometry quantification of the EV staining on GSCs and DGCs showed significant differences between GSCs stained with EV compared to GSCs stained with NT (p=0.0004), DGCs stained with EV (p=0.0028), and DGCs stained with NT (p=0.0011) (**Figure 2C**). The evaluation of the peptide binding affinity by flow cytometry demonstrated a higher affinity of the peptide EV for GSCs compared to DGCs, with a KD of 32.3 μM for GSCs, and a higher KD of 220.7 μM for DGCs (**Figure 2D**). To determine the specificity of the EV peptide to target only glioblastoma, mice bearing intracranial T387 or T4121 xenograft GBM models, or mice that received a sham brain tumor implantation, were injected with 20 μM of EV. Fluorescence imaging demonstrated that only mice with intracranial xenografts had an increased fluorescence signal after peptide injection located only in the brain (**Figure 2E**). The mice with no brain tumors did not show any fluorescence localized in the brain after EV peptide injection.

### EV peptide specifically binds to N-cadherin in GSCs

**Figure 3:**
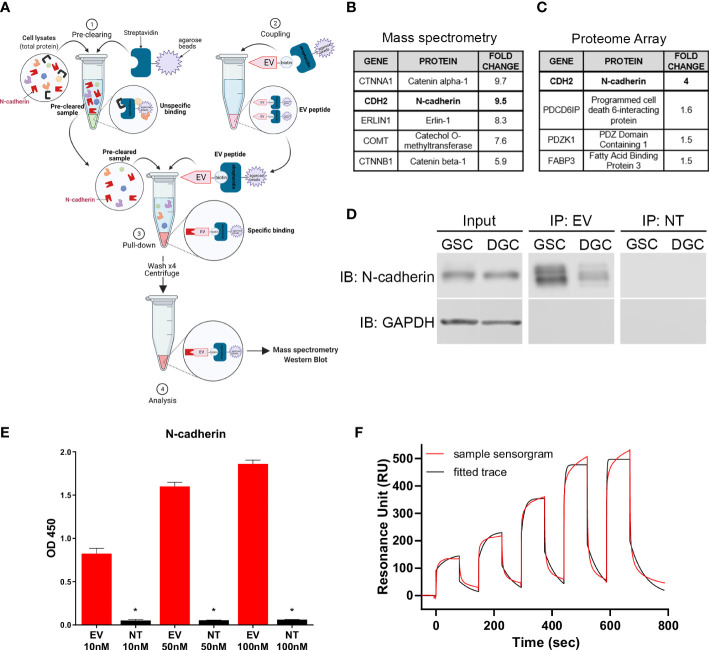
N-cadherin as the target of EV peptide. **(A)** Diagram of the peptide pull-down assay performed with EV-biotinylated peptide on GSCs and DGCs. **(B)** Highest scoring proteins from EV binding to GSC surface proteins identified by peptide pull-down followed by mass spectrometry analysis and **(C)** human proteome array. **(D)** Immunoblot results from the peptide pull-down, demonstrating a stronger binding to N-cadherin in GSCs compared to DGCs. **(E)** ELISA displaying the binding of different concentrations of EV or NT to N-cadherin. The stars show the significant differences between EV and NT for the same concentration, * = p < 0.005. Data in quadruplicate. **(F)** Surface plasmon resonance single cycle kinetic titration run showing the binding of EV peptide with N-cadherin using Biacore T200. Data were performed in duplicate.

A peptide pull-down was performed with EV and NT peptides following incubation with GSC lysates to identify cellular binding partner(s) of EV (**Figure 3A**). Proteins that were collected through the pulldown were analyzed by mass spectrometry. Proteins isolated with EV were compared to proteins isolated with NT to determine the EV binding specificity. The mass spectrometry showed a 9.5-fold increase binding of EV to N-cadherin (CDH2) compared to NT on GSCs (**Figure 3B**). In parallel, binding of EV and NT to the HuProt Human Proteom Microarray (CDI Laboratories) was analyzed. In this analysis, N-cadherin showed a 4-fold increase binding of EV compared to NT peptide (**Figure 3C**).

Immunoblot performed with pulldown samples demonstrated positive signal with N-cadherin antibody in the EV peptide immunoprecipitates while absent in the NT peptide pulldown (**Figure 3D**). N-cadherin was also found to be present in a higher level in GSCs compared to DGCs. The binding affinity of EV to N-cadherin was confirmed by ELISA, which demonstrated dose-dependent binding with increasing concentration of EV to recombinant extracellular N-cadherin protein. All concentrations showed a strong affinity between EV and N-cadherin, compared to NT and N-cadherin (p ≤ 0.0348, **Figure 3E**). Binding affinity evaluation was performed to rule out other binding partners isolated in the mass spectrometry and proteome array analysis to confirm binding to N-cadherin. Binding of EV and NT peptides to CTNNA1 or CTNNB1 in ELISA demonstrated no preference in binding of EV peptide over the NT peptide (**Supplemental Figures 4A, B**).

Surface plasmon resonance was also used to confirm the binding of EV peptide and N-cadherin *in vitro*. The single cycle kinetic results showed a very well fitted response curve by Biacore T200 Evaluation Software to derive an on-rate (k_a_) of 2615 M-1 s-1 and an off-rate (k_d_) of 0.01957 s-1, resulting in an equilibrium binding constant KD of 7.50 µM (**Figure 3F**). A repeated run under the same condition on another new CM5 sensor chip was fitted and resulted in a KD of 9.40 µM, giving an average KD of 8.45 µM. The NT peptide did not exhibit significant resonance units compared with those of the EV and could not be fitted for reliable extraction of the KD. The interaction of EV with N-cadherin was confirmed by flow cytometry after a knockdown of CDH2 in GSCs (**Supplemental Figure 5**).

### CARs using tandem short peptides as the antigen-binding domain recognize GSCs

**Figure 4:**
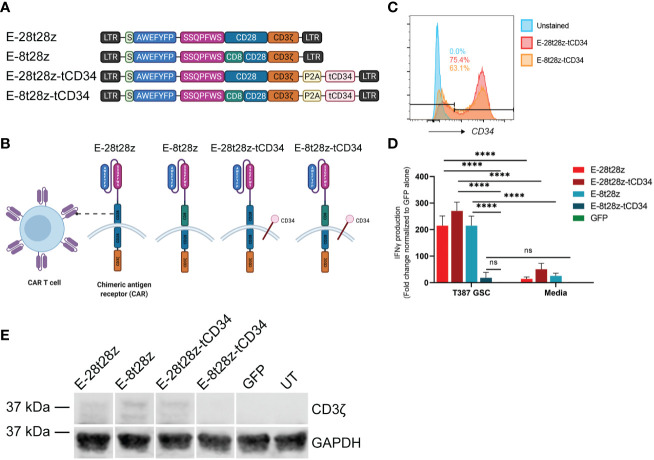
Dual peptide CAR-T cell design. **(A)** Diagram of the CAR construct designs. The E-28t28z and E-8t28z constructs use CD28 and CD8 as the hinge/transmembrane domains respectively, while both constructs employed CD28 for the co-stimulatory domain. The E-28t28z-tCD34 and E-8t28z-tCD34 constructs are identical to the E-28t28z and E-8t28z constructs but feature co-expression of truncated CD34 as an extracellular marker. **(B)** Diagram of a CAR-T cell with the different dual peptide-based constructs as the antigen-recognition domain. **(C)** CD34+ expression analysis by flow cytometry allows for a built-in quantification of transduction efficiency. **(D)** ELISA results displaying IFN-gamma secretion from CAR- or mock-transduced (GFP) T cells co-cultured overnight against T387 GSCs or cell-free media, **** = p < 0.0001. Values shown in the bar chart represent the average of two independent experiments. **(E)** CD3ζ western blotting with GAPDH as a housekeeping gene, used for additional support to confirm the presence or lack of the CAR following transduction. LTR, Long terminal repeat; S, signal peptide; P2A, Porcine teschovirus-1 2A self-cleaving peptide; tCD34, Truncated CD34.

To determine the most efficacious design for our CAR-T cells, EV peptide was inserted in place of the scFv region for various CAR constructs (**Figure 4A**). The E-28t28z construct is comprised of a CD28 hinge/transmembrane domain, a CD28 co-stimulatory domain, and the intracellular signaling domain CD3ζ, while the E-8t28z construct instead contains a CD8 hinge/transmembrane domain. The E-28t28z-tCD34 and E-8t28z-tCD34 constructs are identical to the constructs described above but were co-expressed with a truncated CD34 (tCD34, used here as a cell-surface marker (25)), separated by a P2A cleavage site (**Figures 4A, B**). Staining of these CAR-T cells with anti-CD34-PE antibody allowed for the quantification of transduction efficiency by flow cytometry, showing robust cell transduction (75.4% and 63.1% for the E-28t28z and E-8t28z constructs, respectively, in the representative example shown, **Figure 4C**).

We next analyzed the ability of these CAR-T cells to recognize GSCs. To that end, we co-cultured (CAR-)T cells with GSCs or media alone overnight, and quantified the presence of IFN-gamma in the supernatants. All CAR-transduced T cells (except for E-8t28z-tCD34) showed a significant increase in IFN-gamma produced in response to co-culture with T387 GSCs when compared with mock-transduced (GFP) cells and with each respective CAR in presence of cell-free media (p<0.0001) (**Figure 4D**). To determine the cause of lack of reactivity by E-8t28z-tCD34, we analyzed the expression of the CAR molecules by Western blot. An anti-CD3ζ antibody was used to identify the band corresponding to the CARs, which displayed a higher molecular weight than the endogenous CD3ζ. As shown in **Figure 4E**, the E-8t28z CAR was expressed when cloned in absence of tCD34, but its expression was impaired when co-expressed with the marker gene (full uncut membrane shown in **Supplemental Figure 3**). This finding indicates that the lack of GSC recognition by E-8t28z-tCD34 CAR-T cells observed in **Figure 4D** was due to abrogation of CAR expression. We therefore focused the rest of our experiments on the E-28t28z design.

### Dual peptide CAR induces apoptosis of GSCs

**Figure 5:**
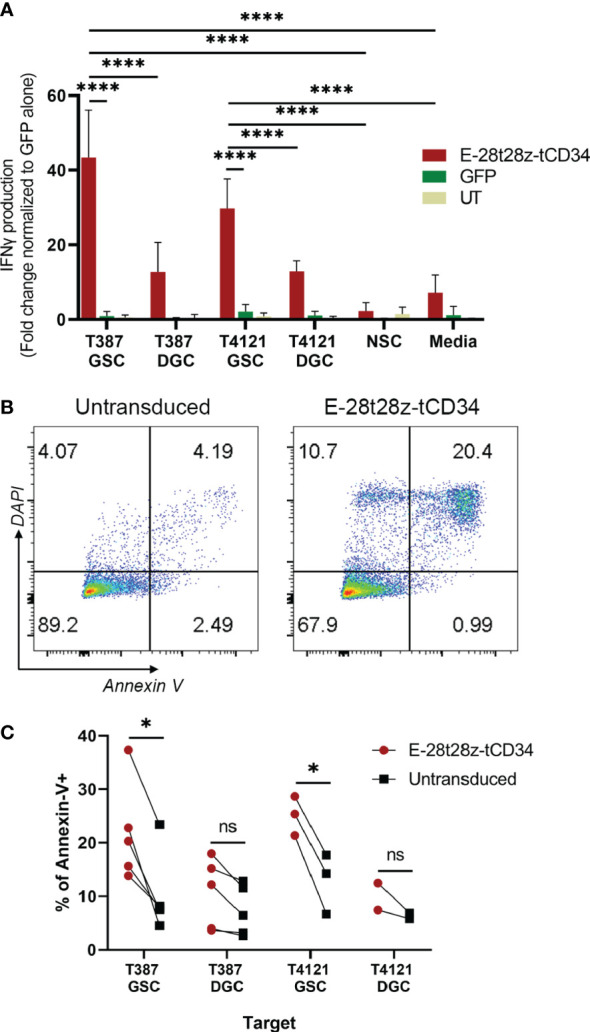
*In vitro* efficacy of the EV CAR-T cells. **(A)** IFN-gamma secretion results from the E-28t28z-tCD34 CAR-transduced, GFP-transduced and untransduced (UT) T cells after overnight co-culture with different cells, **** = p < 0.0001. **(B)** Representative dot plot of Annexin-V staining of GSCs after 4-6 hour co-culture with untransduced and E-28t28z-tCD34 cells. **(C)** Summary of percentage of Annexin-V+ cells following co-culture with the indicated effectors. Target cells include GSC and DGC from two independent donors: T387 and T4121. *p < 0.05 (T387 GSC, and T4121 GSC). n.s.= non statistically significant.

We next evaluated if the selectivity of peptide binding to GSCs over DGCs was preserved in our CAR-T cells. In this experiment, we used two pairs of GSCs/DGCs, each pair derived from the same donor (T387 or T4121). In addition, due to the expression of CDH2 in healthy neural tissue, we tested if the E-28t28z selectively recognizes GSCs over non-malignant neural stem cells. To that end, E-28t28z-tCD34 engineered CAR-T cells were co-cultured with the abovementioned targets. Untransduced/mock-transduced T cells were used as negative controls, as well as CAR-T cells in media alone. CAR-transduced T cells secreted significantly more IFN-gamma in response to co-culture with either T387 and T4121 GSCs when compared with DGCs, NSCs, and cell-free media (p<0.0001). Additionally, CAR-transduced T cells demonstrated a significant increase in IFN-gamma produced in response to co-culture with either T387 and T4121 GSCs when compared with GFP-transduced T cells co-cultured with the same target cell type (p<0.0001) (**Figure 5A**). Although basal reactivity against DGCs was observed, our results confirm that the selectivity for GSCs is preserved. Most importantly, no recognition of NSCs was observed, suggesting that non-malignant stem cells would not be recognized by this CAR.

Finally, we tested the cytotoxic potential of E-28t28z-tCD34 CAR-T cells towards GSCs using Annexin-V staining (**Figure 5B**). Effector and target cells were cultured for 4-6 hours. We then quantified the percentage of Annexin-V-stained cells, in an attempt to capture both early (Annexin-V+/DAPI-) and late (Annexin-V+/DAPI+) apoptotic cells (gating strategy shown in **Supplemental Figure 6**). The assay demonstrated a significant increase of apoptosis induced to T387 (p=0.0404) and T4121 (p=0.0223) GSCs by E-28t28z-tCD34 CAR-T cells when compared to UT T cells, indicating that CAR-T cells can not only recognize but also eliminate GSCs. This difference was not observed in DGCs, reinforcing the notion of selectivity for cancer stem cells (**Figure 5C**).

## Discussion

In this study, we employed peptides that were isolated from previous phage display biopanning (22, 23) with the aim of testing the hypothesis that these short synthetic peptides can be used as antigen-binding domain to augment targeting of GSCs. Establishment of new therapies that will more effectively target and treat GBM must cross the BBB, target tumor cells without damaging the surrounding environment, and kill GSCs to prevent tumor relapse following standard of care treatment (13). These constraints have long served as obstacles against the development of effective interventions for primary malignant brain tumors.

Phage display biopanning has proven effective for the development of targeted therapeutics by isolating peptides that have preferential binding capacity for a given receptor or cell type (26). Extensive literature reports the application of peptides that specifically bind to different types of cells, such as tumor blood vessels, tumor lymphatic vessels, tumor cells, or to specific cell surface receptors, such as integrin and neuropilin, nestin, VPAC1 receptor, FGF9 in gastric and bladder cancer, or CD133 in glioblastoma (27–33). Based on the potential for using phage display biopanning to discover new therapeutic targets, multiple studies have conjugated their discovered peptides with nanoparticles, drugs or antisense oligonucleotides (34–38).

To focus on the targeting of GSCs, a subset of the tumor population which may be heavily involved in resistance to standard therapy, we have previously employed *in vitro* and *in vivo* phage display biopanning strategies to isolate peptides that specifically target and bind GSCs. Phage display biopanning can be used to isolate peptides that mimic a known ligand to replicate a specific ligand-receptor interaction. However, as definitive markers that specifically define GSCs remain elusive, we devised biopanning strategies to select for peptides that may target cellular receptors that are unique to the stem cell phenotype within GBMs. Our goal was then to leverage the specificity of these peptides to augment strategies for therapeutic delivery to GBM specifically by targeting GSCs.

We demonstrated that by combining two different peptides displaying GSC-targeting abilities, we maintained their specificity toward GSCs, and were also able to identify the targeted receptor, N-cadherin (CDH2). N-cadherin is an adhesion molecule with over 96% homology between human and mouse, which involved in the development of neural tissue and in various neurodegenerative processes (39). By using the dual peptide as the extracellular antigen-recognition domain of CAR constructs, we were able to create CAR-T cells that recognized GSCs *in vitro*. Extensive efforts to develop CAR-T cells targeting GBM have been made these past few years, and *in vitro* studies against GSCs with CAR-T cell therapies have demonstrated promising results (8, 40). The most studied tumor-associated antigen targets for CAR-T cells in GBM include interleukin-13 receptor alpha 2 (IL-13Rα2), EGFRvIII, and human epidermal growth factor receptor 2 (HER2) – molecules that can be expressed by GSCs (7, 8, 41). Therefore, CAR-T cell therapy represents an opportunity to eradicate this population of cells, largely implicated in treatment resistance and tumor relapse.

Our study has illustrated the utility and feasibility of designing novel antigen-binding domains for CAR-T cells by using two short peptides of just seven amino acids each to induce functional recognition of GSCs. We evaluated the expression and reactivity of several different EV-based CAR constructs in alpha-beta T cells, narrowing it down to use of CD28 or CD8 as the hinge/transmembrane domains and CD28 as the superior costimulatory domain, with our final CAR constructs exhibiting specific reactivity and cytotoxicity against GSCs with the dual peptide used in the place of the single-chain variable fragment (scFv) as the antigen-binding domain. By using mass spectrometry and human proteome analysis, we were able to isolate N-cadherin as the likely target of the peptide-based recognition of GSCs. N-cadherin is a transmembrane protein that plays a key role in cell-cell adhesion in the brain, and is involved in the epithelial-mesenchymal transition, with increase of its expression while E-cadherin expression is decreased (42–44). N-cadherin has a role in tumor invasion and is known to be expressed in GBM and GSCs (45–48). The expression of N-cadherin has also been associated with WHO glioma grading, correlating with a decreased patient survival when overexpressed (20). The study of Osuka et al. showed that a knockout of N-cadherin by CRISPR/Cas9 reduced the stemness and the resistance of GSCs to radiation therapy (49). Therefore, N-cadherin may be a viable target that allows specific targeting of GSCs. It is unclear at this time as to the exact region of N-cadherin that is targeted by the peptides. We do know that based on the recombinant N-cadherin (reference 1388NC050, R&D Systems Inc., Minneapolis, MN) used for the ELISA binding experiments (**Figure 3E**), that the EV peptide is binding to the extracellular domain between amino acids 160-724. We have previously tested the binding of peptide AWEFYFP (E) and found that by itself it demonstrated positive binding to N-cadherin (manuscript under review). Further studies will be needed to better understand how each peptide affects the binding of peptide EV to N-cadherin.

Despite the specificity of the dual peptide-based CAR-T cells for targeting GSCs over DGCs or NSCs, the targeting of N-cadherin can have some limitations. This protein is widely expressed in normal tissues, particularly in the brain, endocrine tissues, and muscle tissues (especially in the heart). Though we have demonstrated a lack of EV peptide targeting of non-neoplastic brain tissue, further studies are necessary to understand the role of N-cadherin in EV peptide specificity for the targeting of GSCs. However, the knowledge that we have advanced in producing a functional antigen-recognition domain with tandemly arranged short peptides derived from phage-display biopanning could prove useful for the generation of new tethering or costimulatory receptors. Such receptors can serve as a physical bridge linking tumor cell and T cell, increasing functional avidity, and stabilizing or enhancing the immunological synapse without resulting in an activation cascade on their own (50). On-target off-tumor toxicity remains a hurdle in CAR-T cell therapies, though the employment of a Boolean logic “AND” gate in T cell immunotherapy can circumvent this issue by requiring gene-edited T cells to recognize two or more antigens to result in effector function (51, 52). A potential dual-receptor strategy for GBM could involve an “AND” gate approach employing the GSC-targeting peptides in conjunction with other known GBM-targeting CARs.

Further studies utilizing GSC-targeting CAR-T cells will need to be tested to demonstrate the effectiveness of these cells in the *in vivo* setting. Given the potential ubiquity of N-cadherin receptors, different delivery methods may need to be tested, such as intrathecal or intratumoral delivery, to avoid non-specific binding outside of the central nervous system. As GSC-targeting CAR-T cells will be directed against a subset of the GBM tumor population, detailed studies will need to be constructed to safely and effectively deliver the CAR-T cells and to test their effectiveness on limiting on tumor recapitulation and invasion.

## Conclusion

Our results exhibit the potential for utilizing *in vitro* and *in vivo* phage display biopanning approaches to obtain peptides that can serve as the antigen-binding domain of CAR constructs. Our CAR-T cells retained the same specificity toward GSCs as our initial peptides and led to functional reactivity and cytotoxicity. This study demonstrates the potential of developing novel strategies for the treatment and diagnosis of different tumors currently incurable.

## Data availability statement

The datasets presented in this article are not readily available because sharing of materials and data are subject to review and approval by Moffitt’s Office of General Counsel and Office of Innovation. Some materials are bound to MTA. Requests to access the datasets should be directed to james.liu@moffitt.org.

## Ethics statement

The animal study was reviewed and approved by USF IACUC (Protocol RIS00010727).

## Author contributions

DA-D and JL jointly contributed to conception and design of the study. MP, SS, CS, JK, KT, TT, and MR contributed to experimental design and acquisition/analysis/interpretation of data. MP and SS wrote the first draft of the manuscript. All authors contributed to manuscript revision, read, and approved the submitted version.
