# Supplementary material for: Use of phage display biopanning as a tool to design CAR-T cells against glioma stem cells
Source: Front Oncol. 2023 Mar 24;13:1124272. doi: 10.3389/fonc.2023.1124272 (PMC10080078; doi:10.3389/fonc.2023.1124272)
Supplement: Supplementary file 1 [file DataSheet_1.docx]

**Supplemental Methods**

**Mass spectrometry**

Protein disulfides were reduced with 4.5 mM DTT at 60 °C for 30 minutes and then cysteines were alkylated with 10 mM iodoacetamide for 20 minutes in the dark at room temperature. Trypsin digestion was carried out overnight, and tryptic peptides were acidified with aqueous 1% trifluoroacetic acid (TFA) and desalted with C18 Sep-Pak cartridges according to the manufacturer’s procedure.

A nanoflow ultra high performance liquid chromatograph and nanoelectrospray orbitrap mass spectrometer (RSLCnano and Q-Exactive plus, Thermo) were used for LC-MS/MS. The sample was loaded onto a pre-column (C18 PepMap100, 2 cm length x 100 µm ID packed with C18 reversed-phase resin, 5 µm particle size, 100 Å pore size) and washed for 8 minutes with aqueous 2% acetonitrile and 0.1% formic acid. Trapped peptides were eluted onto the analytical column, (C18 PepMap100, 25 cm length x 75 µm ID, 2 µm particle size, 100 Å pore size, Thermo). A 90-minute gradient was programmed as: 95% solvent A (aqueous 2% acetonitrile + 0.1% formic acid) for 8 minutes, solvent B (aqueous 90% acetonitrile + 0.1% formic acid) from 5% to 38.5% in 60 minutes, then solvent B from 50% to 90% B in 7 minutes and held at 90% for 5 minutes, followed by solvent B from 90% to 5% in 1 minute and re-equilibration for 10 minutes using a flow rate of 300 nl/min. Spray voltage was 1900 V. Capillary temperature was 275 °C. S lens RF level was set at 50. Data-dependent acquisition was performed using Top16 precursors. The resolution for MS and MS/MS were set at 70,000 and 17,500 respectively. Dynamic exclusion was 15 seconds for previously sampled peaks.

**Data Analysis**

Spectra were analyzed and quantitated using MaxQuant (1), followed by IRON (2) normalization against the median sample (iron_generic --proteomics --norm-iron=O2_NT7_3). Additional protein annotation was added from UniProt (3) and GenBank (4). All abundances were log_2_ transformed prior to further analyses, with zero abundances treated as missing data. Up to 2 missed trypsin cleavages were allowed. The mass tolerance was 20 ppm first search and 4.5 ppm main search. Carbamidomethyl cysteine was set as fixed modification. Methionine oxidation were set as variable modifications. Both peptide spectral match (PSM) and protein false discovery rate (FDR) were set at 0.01. Match between runs feature was activated to carry identifications across samples.

Average log_2_ abundances were calculated from the biological replicates within each condition. Log_2_ ratios between conditions were calculated by subtracting average log_2_ abundances. Two-group p-values were calculated using two-sided, unequal-variance Welch's T-tests from the individual biological replicates within each condition. Comparisons were determined to be differentially expressed if all of the following criteria were met: row is assigned to at least one protein from the target species, |log_2_ ratio| ≥ ~0.585 [log_2_(1.5-fold)], and p-value < 0.05. Scores were calculated as the geometric mean of the |log_2_ ratio| and -log_10_(p-value), multiplied by the sign of the log_2_ ratio. Rows were sorted on |Score|, direction of change, and differential expression status to bring the rows with the strongest potential biological signal to the top of the analysis spreadsheet.

**References:**

1: Cox J, Mann M. MaxQuant enables high peptide identification rates, individualized p.p.b.-range mass accuracies and proteome-wide protein quantification. *Nat Biotechnol* (2008) 26: 1367–1372. doi: 10.1038/nbt.1511

2: Welsh EA, Eschrich SA, Berglund AE, Fenstermacher DA. Iterative rank-order normalization of gene expression microarray data. *BMC Bioinformatics* (2013) 14:153. doi: 10.1186/1471-2105-14-153

3: The UniProt Consortium, UniProt: the Universal Protein Knowledgebase in 2023, *Nucleic Acids Research (2023)* 51:D523–31. doi: 10.1093/nar/gkac1052

4: Benson DA, Cavanaugh M, Clark K, Karsch-Mizrachi I, Lipman DJ, Ostell J, et al. GenBank, *Nucleic Acids Research* (2013) 41:D36–42. doi: /10.1093/nar/gks1195

**Supplemental Figures**

**Supplemental Figure 1: Gating strategy for ImageStream acquired data**.


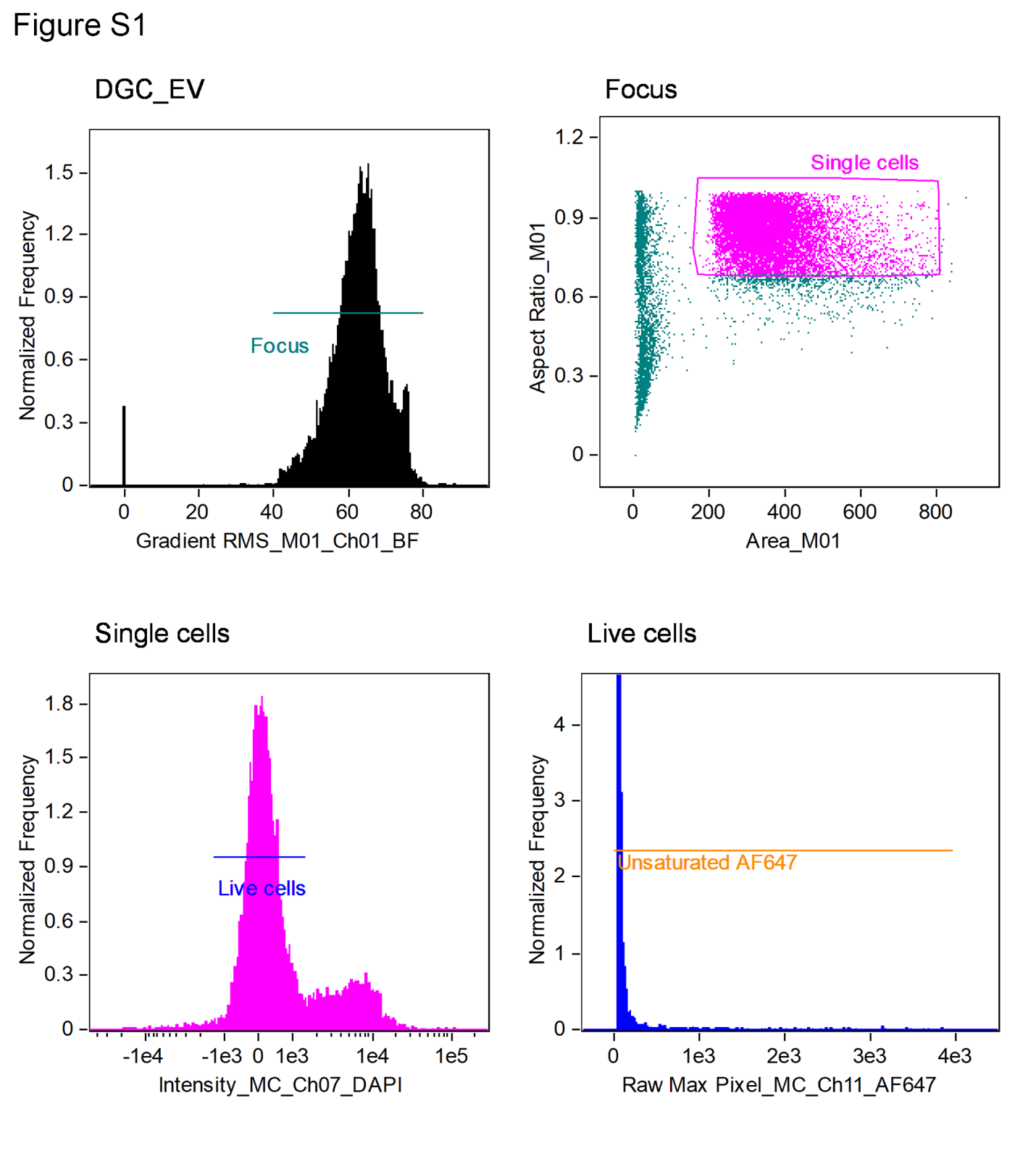


**Supplemental Figure 1: Gating strategy for ImageStream acquired data**. Using the brightfield channel, focused cells are gated, followed by single cells localized between 0.8 and 1 for the Aspect Ratio and between 200 and 800 for the Area. Live cells are gated on the DAPI negative population, and unsaturated cells stained with EV-AF647 (<4×10^3^) are kept for Median Fluorescence Intensity measure.

**Supplemental Figure 2: Immunoblot of EV pull-down.**


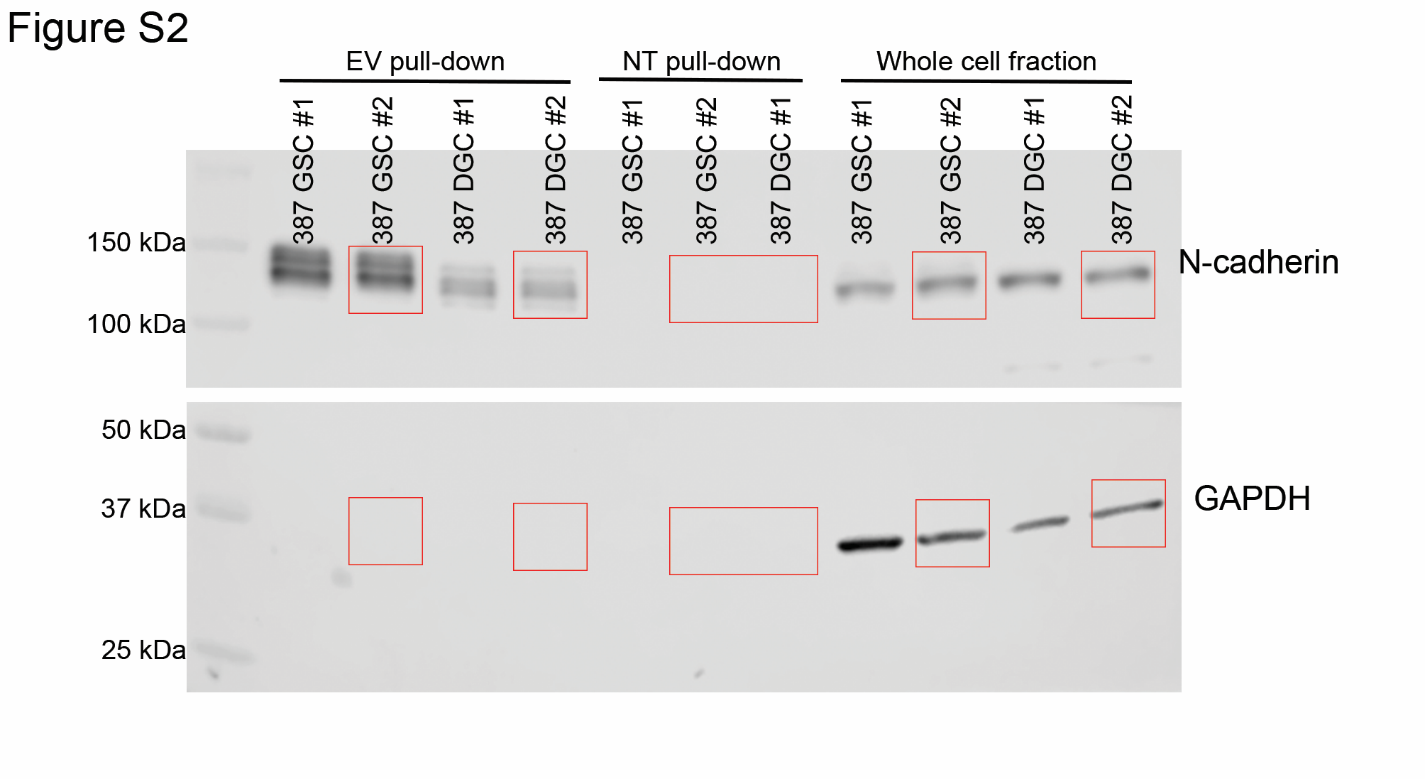


**Supplemental Figure 2: Immunoblot of EV pull-down.** Uncut membranes stained for N-cadherin (top) and GAPDH (bottom). Red squares represent the bands used in Figure 3.

**Supplemental Figure 3: Western blot of CD3ζ and GAPDH.**


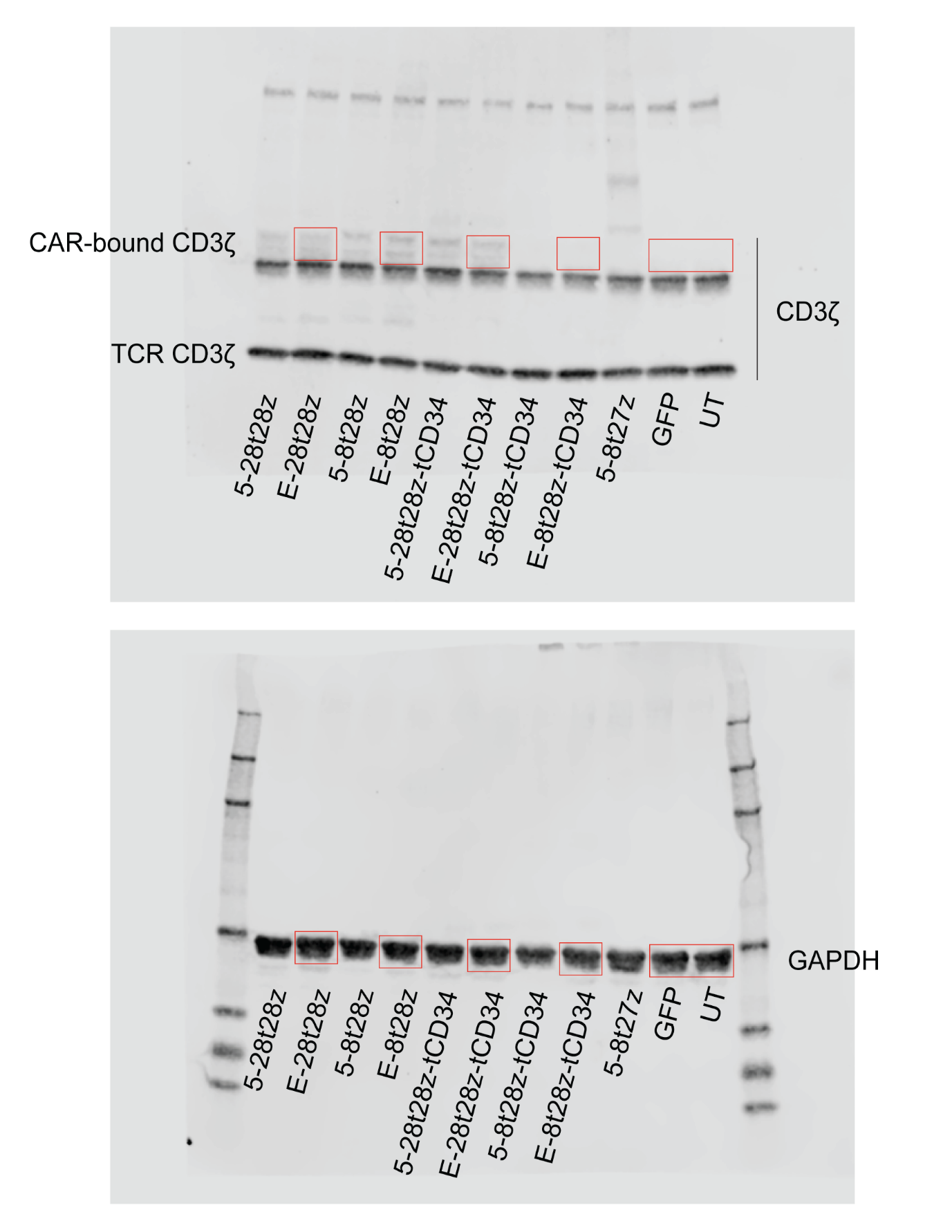


**Supplemental Figure 3: Western blot of CD3ζ and GAPDH.** Uncut membranes stained for CD3ζ (top) and GAPDH (bottom). Red squares represent the bands used in Figure 4.

**Supplemental Figure 4: Binding affinity between EV, CTNNA1 and CTNNB1.**


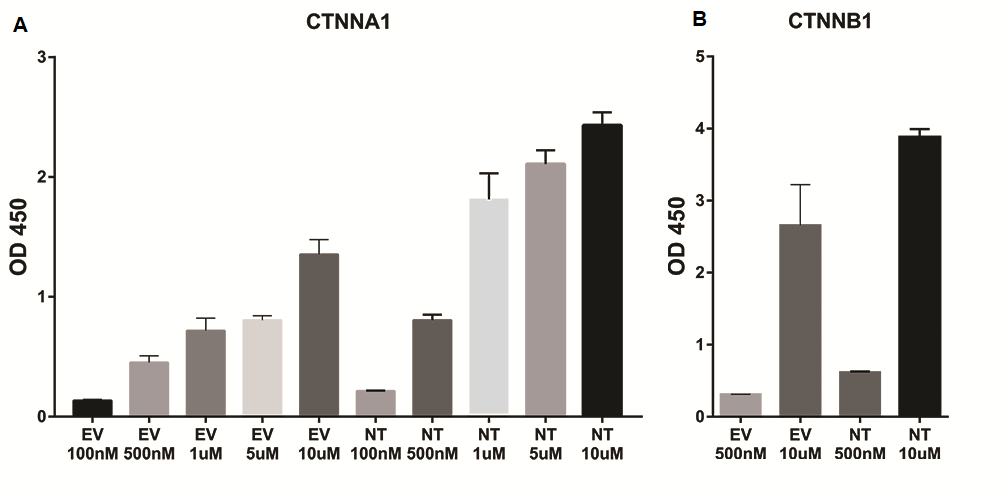


**Supplemental Figure 4: Binding affinity between EV, CTNNA1 and CTNNB1.** ELISA displaying the binding of different concentration of EV or NT peptides to alpha and beta catenin. Data in quadruplicate.

**Supplemental Figure 5: Knockdown of N-cadherin.**


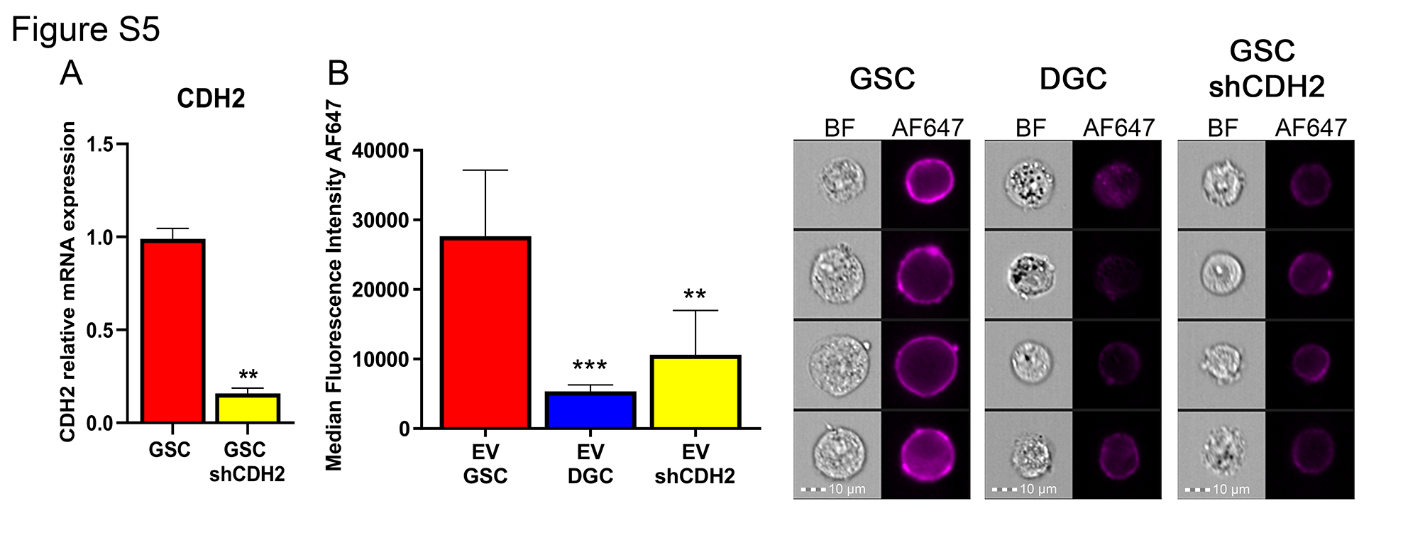


**Supplemental Figure 5: Knockdown of N-cadherin. (A)** RT-qPCR showing the efficiency of CDH2 knockdown (yellow) in GSCs compared to untransduced GSCs (red). The stars represent significance between GSC and shCDH2 GSC, ** = p<0.01. **(B)** Flow cytometry analysis and representative images showing a decreased staining of DGCs (blue) and shCDH2 GSCs (yellow) cells with the EV peptide compared to untransduced GSCs (red). The stars represent significant differences with EV GSC, ** = p<0.01, *** = p<0.001.

**Supplemental Figure 6:** **Annexin-V gating strategy.**


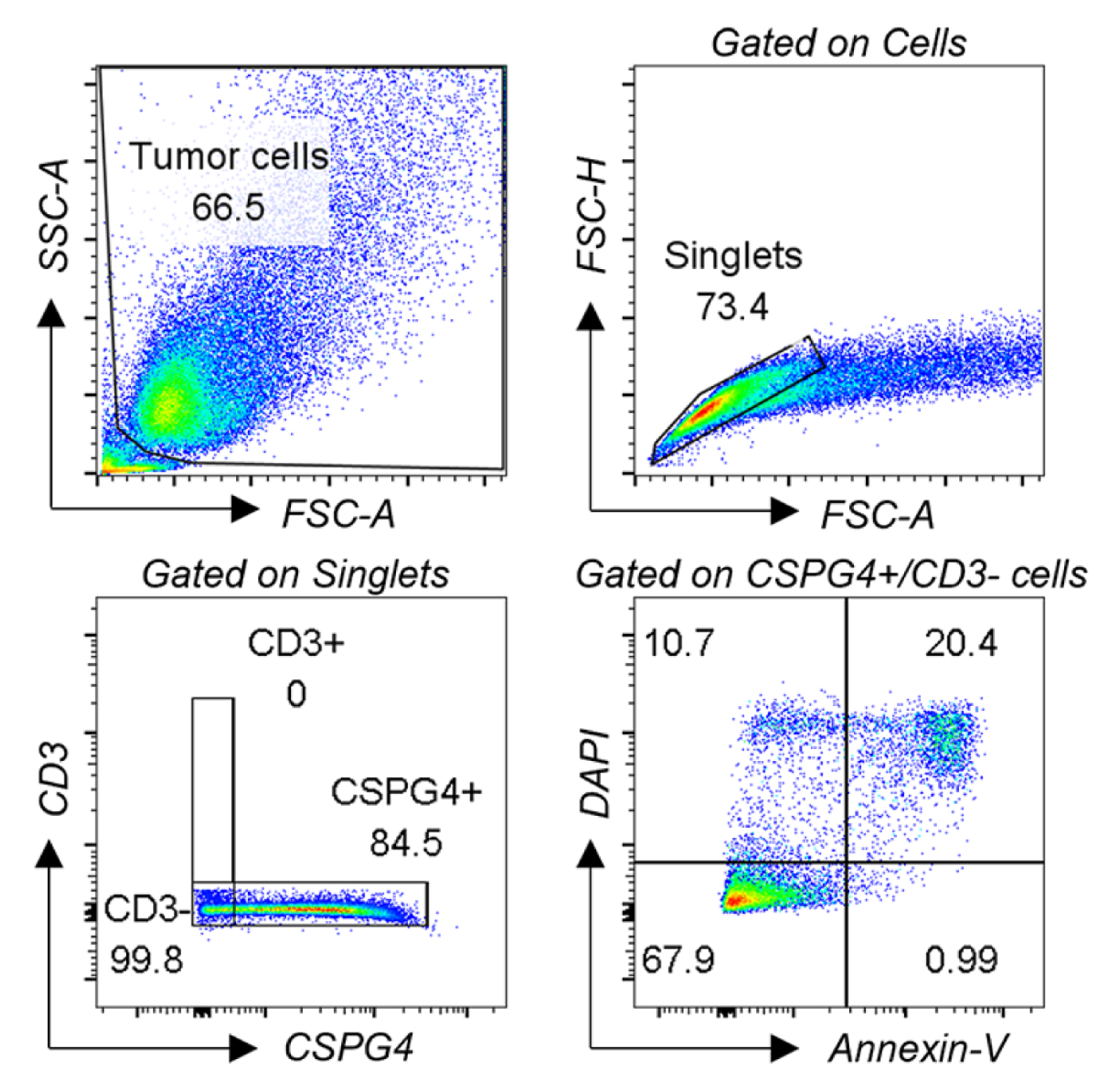


**Supplemental Figure 6:** **Annexin-V gating strategy.** Initial gating was implemented around the tumor cell population, then singlets, then CSPG4+/CD3-, and finally Annexin-V+.
